# Current Endodontic Practices among Romanian Dental Practitioners: A Cross-Sectional Study

**DOI:** 10.3390/dj12090283

**Published:** 2024-09-03

**Authors:** Cezar Tiberiu Diaconu, Lelia Mihaela Gheorghiță, Anca Elena Diaconu, Mihaela Jana Țuculină, Alexandru Gliga, Carlo Gaeta, Simone Grandini, Iulia Roxana Marinescu, Marina Olimpia Amărăscu, Oana Andreea Diaconu

**Affiliations:** 1 Department of Endodontics, Faculty of Dental Medicine, University of Medicine and Pharmacy of Craiova, 200349 Craiova, Romania; diaconu_czr@yahoo.com (C.T.D.); leliagheorghita@yahoo.com (L.M.G.); anka.diaconu@gmail.com (A.E.D.); oanamihailescu76@yahoo.com (O.A.D.); 2 Department of Operative Dentistry, Faculty of Dental Medicine, “Carol Davila” University of Medicine and Pharmacy, 050474 Bucharest, Romania; dr.alexandrugliga@gmail.com; 3 Unit of Endodontics, Department of Medical Biotechnologies, Periodontology, Restorative and Pediatric Dentistry, University of Siena, 53100 Siena, Italy; odontoiatriagaeta@libero.it (C.G.); simogr@gmail.com (S.G.); 4 Department of Rehabilitation Oro-Dental, Faculty of Dental Medicine, University of Medicine and Pharmacy of Craiova, 200349 Craiova, Romania; marinaamarascu@yahoo.com

**Keywords:** endodontics survey, biomaterials, bioceramics, laser technology

## Abstract

Root canal therapy (RCT) is usually performed in Romania by general dentists (GDPs) because they are more readily available and more reasonably priced than endodontists. Concerns have been raised about the quality of RCTs performed by GDPs, possibly due to insufficient equipment or knowledge; therefore, this study aims to investigate current endodontic practices in Romania. Materials and Methods: A cross-sectional study was conducted via a questionnaire distributed to 400 randomly selected Romanian dentists, with 285 fully validated completed responses (71.25% response rate). Respondents were grouped by speciality, and statistical analysis, including cross-tabulation and the χ^2^ (chi-square) test, was used, with a significance level set at *p* ≤ 0.05 for all tests. Results: Significant differences were found in the use of magnification, rubber dam, sodium hypochlorite concentration, and bioceramics (*p* < 0.001). No significant differences were observed in preferences for measuring working length (*p* = 0.166) or rotary instrument motion (*p* = 0.289). Approximately 6% of the respondents used laser technology with no significant difference across specialities (*p* = 0.571). Additionally, 77.9% preferred using sodium hypochlorite, with no significant difference between groups (*p* = 0.006) regarding concentration. *Conclusions*: Most participants, including GDPs, use modern equipment and techniques during RCT, indicating their awareness and competence in current endodontic practices.

## 1. Introduction

The major objectives of root canal therapy (RCT) are to keep the tooth healthy and functioning normally, address the pulp’s inflammatory response to a particular irritating factor, and prevent and treat periapical tissue ailments [1]. Essentially, the goal of RCT is to reduce the number of microorganisms in the root canal system and avoid reinfection by using appropriate chemo-mechanical instrumentation and a tight seal final obturation of the root canal space [2,3]. Over the years, several studies have addressed the topic of success rates in endodontics and found that the overall success rate of RCT varies considerably [4,5,6], from as high as 90% if the RCT is performed by an endodontist [7] to a rate of 65–75% [8] if the treatment is completed by a general dental practitioner (GDP).

In order to quantify the success or failure of RCT, we must divide the endodontic procedure into stages and analyze those that can influence the outcome of RCT, such as the complexity of the root canal system, the expertise of the physician, and the equipment at their disposal. A clinician without proper expertise or equipment might find the identification and enlargement of canals, establishing and maintaining the correct working length, selecting the correct size of preparation for effective irrigation of the root canal system, and adequate obturation challenging [9]. In addition to the principles and treatment procedures that have been evolving over the years and transforming the practice of endodontics, an array of new tools, materials, and techniques have been developed to address these issues [10,11]. The utilization of rubber dam isolation, magnification, and cone beam computed tomography (CB-CT) analysis has evolved into the accepted standard for RCT today [12,13,14], although there are some educational implications regarding the appropriate applications of CBCTs, its interpretation, and access for use [15,16]. Although it is assumed that most clinicians have easy access to the internet and information, enabling the use of electronic knowledge databases that can assist administrators of healthcare systems and clinicians to make evidence-based decisions about the acquisition, usage, and deployment of equipment [17], we believe that academic institutions and dental schools should prepare their students to follow the guidelines and proposed requirements for root canal debridement, shaping, and obturation in accordance with the most recent RCT standards. These have been described in the recommendations for endodontic treatment—Treatment of pulpal and apical disease: The European Society of Endodontology (ESE) S3-level clinical practice guidelines [18]—in order to improve the quality of clinical practice. Such recommendations must be followed by dental practitioners regardless of their speciality. As RCT in many places in Romania is carried out by GDPs, because they are more readily available and more reasonably priced than endodontists, there have been questions as to whether RCT performed in Romania is substandard due to a lack of equipment or knowledge. Therefore, the aim of this study was to investigate the current endodontic practices in Romania and to determine the level of expertise, the equipment available for endodontic practices, and personal preferences regarding the RCT procedures of the Romanian practitioners.

## 2. Materials and Methods

The sample size was determined using the Id Survey Sample Size Calculator (https://www.idsurvey.com/en/sample-size-calculator/, accessed on 1 March 2024), with the confidence level set at 90%. The minimum recommended number of respondents was 267 in order to satisfy the requirement for analysis in a cross-sectional study.

This cross-sectional study was conducted among dentists practicing in Romania through a questionnaire comprising 23 multiple-choice questions. The questionnaire was developed by three faculty members from the Department of Endodontics at the Faculty of Dental Medicine, Craiova, Romania, each with over 15 years of professional experience in dentistry. The questions were designed to monitor a series of primary and secondary variables, providing a comprehensive understanding of the current state of endodontic practice among Romanian dentists. After the questionnaire was checked by a statistician for common errors such as double-barreled, confusing, and leading questions, the questionnaire was disseminated to all participants of two national dental conferences that took place in two different cities in Romania with the help of the organizing committees. In total, 400 dental practitioners were invited to anonymously participate to this survey using Google Forms (Google LLC, Montain View, CA, USA).

The questionnaire remained open for responses for a three-month period, from March to May 2024. Initially, 288 completed questionnaires were obtained; however, due to our exclusion criteria, only 285 fully validated responses were retained. The exclusion criteria were as follows:

- Incomplete responses to all questions.
- The responses were received outside the mentioned time frame.

To ensure an unbiased interpretation of the collected data, members from both the Unit of Endodontics, Department of Medical Biotechnologies, Periodontology, Restorative and Pediatric Dentistry, University of Siena, Italy, and the Department of Endodontics, Faculty of Dental Medicine, University of Medicine and Pharmacy of Craiova, Romania, verified the data’s accuracy. They categorized the respondents into three groups: endodontists (ED), general dentists (NS), and dentists specializing in fields other than endodontology (SOE).

Data for each group were collected, and statistical analysis focused on the following primary variables:

- The time practicing dentistry, to quantify personal experience and knowledge about endodontic procedures.
- Postgraduate training regarding endodontics.
- Number of RCTs performed/week.
- The use of magnification during endodontic procedures.
- The use of a rubber dam.
- The preferred method for measuring the working length.
- The preferred motion of the rotary files.
- Single or multiple use of the rotary files.
- The preferred solution for the disinfection protocol.
- The use of laser technologies in endodontic procedures.
- The use of bioceramics in endodontic procedures.

Secondary variables were also taken into consideration in “The preferred solution for the disinfection protocol” section, namely, the preferred concentration of sodium hypochlorite.

### Statistical Analysis

IBM SPSS Statistics for Windows software, Version 29.0, was used for the statistical analysis of the data (Armonk, NY, USA: IBM Corp.). Nominal data are presented as the absolute frequency and percentage, and continuous variables are expressed as the mean and standard deviation. The association between categorical variables was analyzed using cross-tabulation and the χ^2^ (chi-square) test. If the results of the chi-square test were altered to the extent that they could not be considered, Fisher’s exact test was used. The significance level was set at *p* ≤ 0.05 within all tests.

## 3. Results

This cross-sectional study conducted among dentists practicing in Romania reported 285 fully validated respondents, resulting in a response rate of 71.25%.

When analyzing the time practicing dentistry in order to quantify the personal experience of the respondents, we found that 39.6% had been practicing dentistry for a short time (0–5 years), 22.5% had been practicing for 6–10 years, 24.2% for 11–20 years, and 13.7% had been practicing dentistry for more than 20 years, as shown in Table 1.

When taking into consideration postgraduate training, we found that almost half of the respondents, 48.4%, had no speciality. The rest had as their speciality endodontics (20.7%), general dentistry (19.6%), prosthodontics (8.4%), periodontology (4.9%), orthodontics (3.2%), or pedodontics (0.7%), as shown in Table 2. Moreover, 6% of the respondents had more than one speciality.

Furthermore, when taking into consideration the time practicing dentistry and the respondents’ specialities, we observed that, in the case of respondents specializing in endodontics and those without a speciality, the balance was tilted towards those with less seniority: 42.4% with seniority between 0 and 5 years in the case of endodontists and 45.7% with seniority between 0 and 5 years in the case of respondents with no speciality. Regarding the respondents with specializations other than endodontics, the distribution of seniority in the dental field was balanced: 28.4% had seniority between 0 and 5 years, 25% had seniority between 6 and 10 years, 20.5% had seniority between 11 and 20 years, and 26.1% had more than 20 years of experience, as shown in Table 3.

When taking into consideration the number of RCTs performed weekly, in order to further highlight the personal experience regarding endodontic procedures of each respondent, we noticed that 36.5% of the respondents perform between 0 and 5 endodontic treatments per week, while 28.1% undertake between 5 and 10 treatments, and 27.4% perform more than 10 treatments weekly. Additionally, 8.1% of the respondents reported that they do not conduct endodontic treatments.

When statistical analysis was performed, we noticed a statistically significant difference (χ^2^ = 29,693; *p* < 0,01) between the groups, as seen in Table 4. In total, 49.2% of endodontists stated that they perform more than 10 endodontic procedures/week, while respondents from the Speciality other than endodontics and No speciality groups stated than they perform more than 10 endodontic procedures/week in a much smaller percentage, 12.5% and 26.8%, respectively.

Regarding the use of magnification during endodontic procedures, 21.1% of the respondents used dental loupes, 27.7% used the dental operating microscope, and 9.8% used both. A total of 41.4% did not use any kind of magnification during endodontic treatments, as seen in Table 5.

Further statistical analysis of the data revealed that a significantly higher proportion of practitioners with postgraduate training in endodontics (57.6%) used the dental operating microscope for magnification, compared to the other groups. The respondents with other specialities or those without a speciality often did not use magnification, and among those who did use it, most used dental loupes. Medical speciality was significantly associated (*p* < 0.001) with the use of magnification, as seen in Table 4, to carry out endodontic treatments. Practitioners with postgraduate training in endodontics used magnification in a statistically significantly higher proportion than those without a speciality or with other specialities.

We also found statistically significant differences in the use of rubber dam among the three groups. A total of 86.4% of the respondents with postgraduate training in endodontics always used rubber dam isolation for endodontic procedures, while among the respondents from other specialities, only 43.2% always used dental dam isolation, and among those without a speciality, 63.8% always used rubber dam isolation.

The result of the chi-square test (χ^2^ = 30.483; *p* < 0.001) confirmed the difference between the three groups regarding the frequency of the use of rubber dam. Respondents with postgraduate training in endodontics used this procedure significantly more frequently, as shown in Table 4.

For the preferred method of measuring the working length, no significant differences between the three studied groups were found. The association between the speciality and the preferred method of measuring working length was not statistically significant (χ^2^ = 11.687; *p* = 0.166), as shown in Table 4.

The preferred methods for measuring working length are the apex locator (mentioned by 58.6% of the respondents) and a combination of different measurement methods (apex locator, radiological method, and CB-CT), as mentioned by 36.8% of the respondents, as seen in Table 6.

When asked about the preferred motion of the rotary files, continuous rotation movement was preferred by 47% of the respondents for root canal preparation, while reciprocating movement was preferred by 20.4% of the study participants. A total of 32.6% preferred both methods. When we compared the three groups to see whether there was a particular preference for the movement of the rotary instrument for root canal preparation, we found no significant differences. Therefore, there was no statistically significant association (χ^2^ = 4.982; *p* = 0.289) between the speciality and the preference for a certain movement of the rotary endodontic instrument in root canal preparation, as shown in Table 4.

According to the respondents of the present survey, only 9.8% used the rotary instrument in only one endodontic treatment, as shown in Table 7. The remaining 90.2% used it more than once.

When we compared the three groups to see whether there was a particular correlation between the speciality and the single or multiple use of the rotary instrument, we found no significant differences (χ^2^ = 3.420; *p* = 0.181), as seen in Table 4.

When analyzing the preferred solution for the disinfection protocol, sodium hypochlorite was used by 77.9% of those who answered the questionnaire, while 50.2% used EDTA, 27% saline, and 10.5% chlorhexidine. A total of 30.5% of the respondents used combinations of the above solutions. When statistical analysis was performed the result of the chi-square test (χ^2^ = 4.145; *p* < 0.657), as seen in Table 4, confirmed that there was no significant difference between the three groups regarding the preferred solution for the disinfection protocol.

Furthermore, we found that the vast majority of the respondents of this survey (82.1%) preferred the use of sodium hypochlorite with a higher concentration (5.25%), as shown in Table 8. Moreover, when comparing the three groups, we found no statistically significant differences (*p* = 0.006), as seen in Table 9.

According to the validated responses, only a small percentage of the respondents, 6%, regardless of their speciality, used laser technology in endodontics, while 9.8% reported that they planned to use it in the near future. The other 84.2% did not use this technology, nor did they plan to implement this technology in their daily practice in the near future. Although the percentage of respondents with postgraduate training in endodontics who stated that they used laser technology was slightly higher at 8.5% (Table 10) than among respondents with other specializations, the difference was statistically insignificant (χ^2^ = 2.924; *p* = 0.571), as shown in Table 4. Therefore, endodontists did not appear to use laser technology to a significantly higher extent.

When questioned about the use of bioceramics in their daily practice, 61% of the respondents with postgraduate training in endodontics stated that they preferred the use of bioceramics for root canal obturation, while 39% preferred other types of sealers. The results of the statistical analysis showed a statistically significant difference (χ^2^ = 26.148; *p* < 0.001) between the three groups regarding the preference for root canal obturation sealer, as shown in Table 4.

## 4. Discussion

The present cross-sectional study aimed to evaluate current endodontic practices among Romanian dental practitioners. However, this study has a retrospective character, which allowed the respondents to give subjective answers to the questions. Therefore, these answers may deviate from adequate clinical facts. Furthermore, it must be noted that the results are entirely based on answers from dental practitioners with an interest in this survey; thus, caution must be exercised in generalizing these results among all Romanian dentists.

Root canal treatment (RCT) performed without establishing a correct diagnostic beforehand and without proper instruments, knowledge of the root canal system anatomy, or proper techniques leads to uncertain results. The endodontic treatment of any tooth is a difficult procedure, as its success depends on the precise cleaning, shaping, and obturation of a canal using the appropriate armamentarium and strict asepsis protocol [19]. In the present study, out of 285 respondents, almost half, 48.4%, were general dental practitioners (GDPs) without any postgraduate training in endodontics or any other speciality training. The rest had endodontics (20.7%), general dentistry (19.6%), prosthodontics (8.4%), periodontology (4.9%), orthodontics (3.2%), and pedodontics (0.7%) as their specialities. From this total, based on the question about the number of RCT/week, our study revealed that 8% of the respondents performed only emergency RCT and referred the rest of the patients to endodontists for treatment, while the vast majority of the respondents, 92%, performed RCT themselves, which was in accordance with other studies that found similarly low referral rates [20,21]. Most of the respondents were young practitioners with less than 5 years’ experience in practicing dentistry, while only 13.7% of the respondents had more than 20 years’ experience in the field of dentistry. Furthermore, almost half of the respondents with less than 5 years’ experience had undergone postgraduate training in endodontics; therefore, the interest shown by young practitioners in postgraduate training in endodontics is increasing. This finding was in accordance with a previous study carried out in New Zealand, which also found a high level of interest in endodontics training [22]. The present survey revealed that the respondents preferred to use rubber dams during endodontic procedures. A total of 86.4% of the respondents with postgraduate training in endodontics always used rubber dam isolation for endodontic procedures; this may have been due to the fact that young practitioners might be more accustomed to using rubber dam during endodontic procedures, as it was mandatory during their training, unlike for the respondents who graduated more than 20 years ago. Since rubber dam was first introduced by Dr. Barnum in 1864, it saw a constant increase in usage by dental practitioners worldwide until the present, when its usage has become an integral part of endodontic therapy procedures. Rubber dam usage is encouraged worldwide for the isolation of the operative area, in order to provide an aseptic field, control infection, and prevent the ingestion or aspiration of dental instruments [23].

The present survey found that most of the respondents without a speciality always used rubber dam during endodontic procedures, which was in accordance with other studies [24] that found a positive prevalence of rubber dam usage among general dentists during RCT, showing that the practitioners involved in this study understand its importance and have the necessary skills and tools to employ its usage.

Regarding the use of magnification during RCT, the naked eye can only see clearly up to the level of the pulp chamber floor and the canal orifice [25]. Furthermore, with age, natural vision begins to deteriorate [26,27]. Visual disability related to age can be minimized if some kind of magnification is employed [28,29], making its use important for the outcome of endodontic procedures. In the present survey, 58.6% of the respondents were aware of this visual handicap and were using some kind of magnification: 21.1% of the respondents used dental loupes, 27.7% used a dental operating microscope, and 9.8% used both. These findings were in contrast with those of a previous study conducted in 2017 in the city of Tirgu Mures, Romania [30], which found that the vast majority of GDPs were not using magnification in their daily practice. This increase in the use of magnification by dental practitioners may be due to the fact that, in the past decade, there has been increased exposure of practitioners to the benefits of utilizing magnification in endodontic procedures. When taking into consideration the type of magnification preferred, we found that the percentages of dental loupes and dental operating microscopes users were in agreement with those found in the other survey studies [31] conducted with GDPs, which found similar percentages of dental operating microscope usage amongst GDPs.

When asked about the preferred method for measuring the working length, most of the respondents preferred the electronic apex locator, and 36.8% of the respondents stated that they used the electronic apex locator in conjunction with other methods of measuring the working length, such as cone beam computed tomography (CB-CT) or periapical radiograph. According to various studies [32,33], this is the most accurate and safe method for measuring the working length. Romanian dental practitioners have the knowledge and ability to adhere to modern endodontic practice guidelines.

For the instrumentation of root canals and the file motion kinematics, continuous rotation movement was preferred by 47% of the respondents for root canal preparation, while reciprocating movement was preferred by 20.4% of the study participants. A total of 32.6% preferred both methods.

When we compared the three groups regarding preference for the movement of the rotary instrument for root canal preparation, we found no significant differences (*p* = 0.289).

Although reciprocal movement is a safer method of instrumentation for root canals according to various studies [34,35], the conventional continuous rotation movement is still preferred by the majority of dental practitioners, most likely due to the fact that reciprocal movement requires a higher degree of specialization, and as students, most of the respondents were not taught about this option. Some studies highlight the positive impact of introducing reciprocal movement in dental school [36]. Considering that the vast majority of the respondents stated that they used endodontic files more than once, it may be beneficial to employ reciprocal movement, since it improves cyclic fatigue performance [35,37]. While multiple cycles of sterilization and use adversely affect the cutting efficiency and surface roughness of the files [35,38,39], the most significant negative impact is the diminished cyclic fatigue resistance. This reduction in fatigue resistance can result in file breakage within the root canal during instrumentation, thereby jeopardizing the outcome of the endodontic procedure [40,41]. Most of the respondents identified sodium hypochlorite as their preferred solution for the disinfection protocol, in accordance with other studies that found similar rates of sodium hypochlorite usage among dentists [42,43]. Furthermore, we found that the respondents to this survey preferred a high concentration of sodium hypochlorite, 5.25%, in accordance other studies [16], probably for the benefits that it brings in collagen dissolution in pulpal tissue [44] and the greater antibacterial effect when compared to mild concentrations (2–2.5%) [45,46]. Sodium hypochlorite must be used with care, especially in conjunction with other irrigants such as EDTA, because the latter reduces the tissue-dissolving capability of sodium hypochlorite due to the loss of free available chlorine [47,48]. Unfortunately, some of the respondents seemed not to be aware of this fact, because when asked whether they used another solution in conjunction with sodium hypochlorite, 30% responded that the preferred solution used with sodium hypochlorite was EDTA.

Furthermore, we found no statistically significant difference (*p* = 0.006) between respondents with postgraduate training in endodontics and the use of sodium hypochlorite at a high concentration compared to respondents from other specialities.

When considering the use of laser technologies in endodontic procedures, which is considered a state-of-the-art practice, we found that only 6% of the respondents use laser technology in their current endodontic practice and only 10% intend to acquire lasers in the near future. This finding was in accordance with other studies that found similar percentages regarding the use of laser technology in endodontics [49]. Although the use of lasers can have certain drawbacks, including the potential for causing thermal injury, following the protocol for root canal irradiation prevents thermal injuries from occurring to the external root walls or the surrounding tissues [50].

One potential obstacle to the widespread use of lasers in clinical dentistry is the requirement that they should only be used by dentists with advanced training, together with the cost factor [51]. Furthermore, although the therapeutic effects of lasers are different depending on the type and operating protocol of each laser, we found no significant differences (*p* = 0.571) when analyzing the three groups, showing that there was no link between the speciality of practitioners and the implementation of laser technology [52]. In conclusion, regarding the implementation of laser technology in daily practice, similar to all other medical specialities, dentistry is constantly evolving in order to improve efficiency and patient comfort. Practitioners have the duty to review protocols and constantly strive to stay up to date in order to guarantee the quality of both the services provided and, most importantly, the patient’s view of the entire dental system [53].

In the present study, although the respondents were reluctant to implement laser technologies in their endodontic practices, we found that they widely adopted and implemented bioceramics. This was probably due to their wide range of applications in endodontic therapy, ease of use, and benefits like antimicrobial activity due to the high pH and release of calcium ions [54,55,56]. The statistical analysis found a statistically significant difference (*p* < 0.001) between the three groups regarding the preference for root canal obturation sealer, thus proving that the respondents to our survey who had postgraduate training in endodontics were more likely to have implemented bioceramics in their practice than respondents with no speciality or with a speciality other than endodontics. The Romanian practitioners’ overall knowledge and implementation of bioceramics were similar to those found in other studies [57], and this is a good indicator that the respondents to this survey were up to date regarding the new techniques and materials developed for the endodontic field.

Cross-sectional studies are designed to observe a group of participants at a specific point in time. However, these studies have certain limitations. In the present study, there is a lack of information regarding the unbiased evaluation of their practices by the respondents. Additionally, this study relies solely on the practitioners’ evaluations of the quality of their own work, without any methods to assess the proper implementation of medical techniques or the proper use of materials and instruments. Therefore, a cross-sectional radiological examination study with follow-ups at specific time intervals would provide valuable insights into the quality of endodontic therapy performed by Romanian practitioners and yield more reliable data. Although this survey has a limited number of respondents, the data gathered are very interesting and can be used to outline and compare the current standard of endodontic practice in Romania. Based on this idea, further research will undoubtedly provide a valuable contribution to the scientific literature.

## 5. Conclusions

This study demonstrated that the majority of practitioners, with or without postgraduate training in endodontics, who perform endodontic procedures employ modern equipment and techniques during RCT, such as rubber dam isolation, magnification, and bioceramics. The practitioners involved in this study understood the importance of the modern armamentarium and had the necessary skills and knowledge to employ its usage. However, despite the critical nature of the disinfection stage in endodontic procedures, the results from this study were not satisfactory in this area, especially regarding the use of sodium hypochlorite in conjunction with EDTA as irrigants, and the fact that some of the respondents were not aware of the downside of these two solutions used in conjunction. Thus, there is a need to regularly check and update the practices used by dental practitioners for endodontic therapy.

## Figures and Tables

**Table 1:** Time practicing dentistry.

Number of Years	Frequency	Percentage (%)
0–5 years	113	39.6
6–10 years	64	22.5
11–20 years	69	24.2
More than 20 years	39	13.7
Total	285	100.0

**Table 2:** Respondent distribution based on postgraduate training.

Speciality	Number of Responses	Percentage (%) of Respondents	Percentage (%) of Responses
Endodontology	59	19.5%	20.7%
No speciality	138	45.7%	48.4%
Orthodontics	9	3.0%	3.2%
Periodontology	14	4.6%	4.9%
Prosthodontics	2	0.7%	0.7%
Pedodontics	24	7.9%	8.4%
General dentistry	56	18.5%	19.6%
Total	302	100.0%	106.0%

**Table 3:** Respondent distribution by number of years of experience and speciality.

Speciality	0–5 Years/Percentage (%)	6–10 Years/Percentage (%)	11–20 Years/Percentage (%)	More than 20 Years/Percentage (%)	Total
Endodontists	25/42.4%	11/18.6%	15/25.4%	8/13.6%	59/100%
Speciality other than endodontics	25/28.4%	22/25.0%	18/20.5%	23/26.1%	88/100%
No speciality	63/45.7%	31/22.5%	36/26.1%	8/5.8%	138/100%
Total	113/39.6%	64/22.5%	69/24.2%	39/13.7%	285/100%

**Table 4:** Statistical analysis of the main variables.

Variable	Pearson Chi-Square			Cramer’s V		Number of Respondents
	Value	df	*p*	Value	*p*	
Number of RCTs performed/week.	29.693	6	0.000	0.228	0.000	285
Use of magnification during endodontic procedures.	67.133	6	0.000	0.343	0.000	285
Use of a rubber dam.	30.483	4	0.000	0.231	0.000	285
Preferred method for measuring the working length.	11.687	8	0.166	0.143	0.166	285
Preferred motion of the rotary files.	4.982	4	0.289	0.093	0.289	285
Single or multiple use of the rotary files.	3.420	2	0.181	0.110	0.181	285
Preferred solution for the disinfection protocol.	4.145	6	0.657	0.085	0.657	285
Use of laser technologies in endodontic procedures.	2.924	4	0.571	0.072	0.571	285
Use of bioceramics in endodontic procedures.	26.148	4	0.000	0.214	0.000	285

**Table 5:** Use of magnification during endodontic procedures.

Type of Magnification	Number of Respondents	Percentage (%)
Dental loupes	60	21.1
Dental operating microscope	79	27.7
Both	28	9.8
No type of magnification	118	41.4
Total	285	100.0

**Table 6:** Preferred method of measuring working length based on speciality.

Speciality	Apex Locator *n*/(%)	Radiological Method *n*/(%)	CB-CT *n*/(%)	Combination of Methods *n*/(%)	None *n*/(%)
Endodontists	39/66.1%	0/0%	0/0%	20/33.9%	0/0%
Speciality other than endodontics	48/54.5%	5/5.7%	1/1.1%	31/35.2%	3/3.4%
No speciality	80/58%	2/1.4%	0/0%	54/39.1%	2/1.4%
Total	167/58.6%	7/2.5%	1/0.4%	105/36.8%	5/1.8%

*n*—number.

**Table 7:** Descriptive statistics for the single or multiple use of endodontic files.

Responses	Frequency	Percentage (%)
Multiple use	257	90.2
Single use	28	9.8
Total	285	100.0

**Table 8:** Descriptive statistics for the preferred concentration of sodium hypochlorite.

Speciality	Percentage of NaOCl 5.25% Users	Percentage of NaOCl 2% Users	Total (%)
Endodontists	83.1%	16.9%	100.0%
Speciality other than endodontics	71.6%	28.4%	100.0%
No speciality	88.4%	11.6%	100.0%
Total	82.1%	17.9%	100.0%

NaOCl—sodium hypochlorite.

**Table 9:** Descriptive statistical analysis of the preferred concentration of sodium hypochlorite.

Chi-Square Test			
	Value	df	*p*
Pearson chi-square	10.386	2	0.006
Cramer’s V	0.191		0.006
Number of responses	285		

**Table 10:** Descriptive statistics for the use of laser technology in endodontic procedures.

Speciality	Yes (%)	No (%)	Intend in the Future (%)	Total (%)
Endodontists	5 (8.5%)	51 (86.4%)	3 (5.1%)	59 (100%)
Speciality other than endodontics	5 (5.7%)	72 (81.8)	11 (12.5%)	88 (100%)
No speciality	7 (5.1%)	117 (84.8%)	14 (10.1%)	138 (100%)
Total	17 (6%)	240 (84.2%)	28 (9.8%)	285 (100%)

## Data Availability

The data from this research are available from the corresponding authors upon reasonable request.
